# Knee implant kinematics are task-dependent

**DOI:** 10.1098/rsif.2018.0678

**Published:** 2019-02-06

**Authors:** Pascal Schütz, Barbara Postolka, Hans Gerber, Stephen J. Ferguson, William R. Taylor, Renate List

**Affiliations:** Institute for Biomechanics, ETH Zurich, Leopold-Ruzicka-Weg 4, 8093 Zürich, Switzerland

**Keywords:** videofluoroscopy, moving fluoroscope, total knee arthroplasty, tibio-femoral kinematics, activities of daily living, gait activities

## Abstract

Although total knee arthroplasty (TKA) has become a standard surgical procedure for relieving pain, knowledge of the *in vivo* knee joint kinematics throughout common functional activities of daily living is still missing. The goal of this study was to analyse knee joint motion throughout complete cycles of daily activities in TKA subjects to establish whether a significant difference in joint kinematics occurs between different activities. Using dynamic videofluoroscopy, we assessed tibio-femoral kinematics in six subjects throughout complete cycles of walking, stair descent, sit-to-stand and stand-to-sit. The mean range of condylar anterior–posterior translation exhibited clear task dependency across all subjects. A significantly larger anterior–posterior translation was observed during stair descent compared to level walking and stand-to-sit. Local minima were observed at approximately 30° flexion for different tasks, which were more prominent during loaded task phases. This characteristic is likely to correspond to the specific design of the implant. From the data presented in this study, it is clear that the flexion angle alone cannot fully explain tibio-femoral implant kinematics. As a result, it seems that the assessment of complete cycles of the most frequent functional activities is imperative when evaluating the behaviour of a TKA design *in vivo*.

## 1. Introduction

Total knee arthroplasty (TKA) has become a standard surgical procedure for relieving pain and restoring function in patients with degenerative joint diseases, mainly osteoarthritis. Although most patients show little or no impairments after surgery, a large number of TKAs still fail in the longer term due to polyethylene wear, loosening, knee instability or infection [1]. In order to better understand the mechanisms leading to early failure or an unsatisfactory outcome in general, kinematic and kinetic measures during functional activities of daily living can provide a crucial understanding for further improving the longevity and functionality of TKAs. Through providing a baseline for developing and validating biomechanical models, such knowledge can allow the improvement of rehabilitation techniques, as well as the development of new concepts for knee implants.

Investigations into TKA function during complete gait cycles using skin-marker-based motion analysis have been successful in determining global segment kinematics, thereby allowing the estimation of external joint moments [2–4]. However, this approach is known to be strongly affected by soft tissue artefacts [5,6] and does not allow an accurate quantification of tibio-femoral antero-posterior (A–P) translation and internal/external rotation [6–9]. In order to determine such inaccessible joint kinematics, imaging methods such as single-plane fluoroscopy [10–12], as well as dual orthogonal fluoroscopy [13,14], with a subsequent two-dimensional/three-dimensional registration, now allow an analysis of the relative movement of the implant components without soft tissue artefact. Since these static fluoroscopic systems possess a limited field of view, they are constrained to capturing only very restricted movements of the knee (e.g. during sitting/standing, deep knee bends) or allow only a small portion of the whole motion cycle to be tracked [15–20]. As a result, these techniques provide little or no access to functional measurement of activities that involve either loading and unloading, toe-off and heel strike impact, or muscle activation and deactivation, and especially not throughout multiple consecutive cycles. To overcome these limitations of a static image intensifier, dynamic single and dual plane systems have been introduced [12,21,22]. The moving fluoroscope developed at the Institute for Biomechanics, ETH Zürich, allows not only tracking of free-level gait but also tracking of the knee joint during stair descent, which is considered to be a challenging daily activity for subjects with knee disorders [3,23].

To allow a sufficient range of motion and avoid overloading of the passive structures [24,25], the kinematic behaviour of the natural tibio-femoral joint has been of high interest, and has therefore been investigated extensively in cadaveric studies [26], using bone-pins [7], as well as MRI [27,28], RSA [29] and videofluoroscopy [30–32]. Similarly, the biomechanical outcomes after TKA have been extensively examined in order to understand the specific design characteristics of the replacement joint that allow healthy knee joint kinematics to be mimicked [26], and thereby avoid overloading of the surrounding soft tissue structures. Despite the high level of interest, the relative motion of the tibio-femoral joint remains controversially discussed, possibly due to the different techniques used to analyse the movement data, which are known to affect the interpretation of the kinematics [33,34]. As an example, both medial and lateral pivot motions in the transverse plane have been found during a similar change in flexion angle [26,32]. However, despite these problems, it is still clear that a number of factors do play a role in modulating joint kinematics. Here, knee flexion angle [26], limb alignment [35] and different design of the implant [36,37] are all known to play critical roles in the biomechanical outcome of the joint, but these have mainly been assessed either quasi-statically or during very restricted movements of the knee. Importantly, knowledge of changes in the motion between the tibia and femur during the most common functional activities of daily living, i.e. walking and stair descent are still missing but could be critical for understanding the dominant biomechanical influences on the joint. Specifically, no data are currently available examining the tibio-femoral kinematics during complete consecutive cycles of both free-level gait and stair descent.

With the aim to establish whether a significant difference in joint kinematics occurs between functional tasks, the tibio-femoral motion was analysed by videofluoroscopy during walking and stair descent, in comparison to sit-to-stand-to-sit, in subjects with a TKA, especially focusing on tibio-femoral internal/external rotation and A–P translation of the condyles.

## 2. Material and methods

### 2.1. Subjects

One female and five male subjects (average age of 72.8 ± 8.5 years; BMI 24.3 ± 2.2 kg m^−2^) with a unilateral PFC Sigma Curved cruciate retaining (CR) fixed-bearing TKA (DePuy Synthes, Johnson and Johnson), provided written, informed consent to participate in this study, which was approved by the local ethics committee (EK 2011-N-6). All subjects exhibited a good functional outcome (KOOS score 91.2 ± 5.7, no/very low pain with a VAS less than 2) and were measured in the gait lab at least 1 year post-operatively (4.2 ± 3.5 years).

### 2.2. Motion tasks

Level walking, stair descent and sit-to-stand-to-sit were measured in this study. Prior to radiographic measurements, trials without imaging were performed until the subjects felt comfortable walking with the moving fluoroscope. For each motion task, five valid cycles were captured, in which the knee remained within the field of view of the image intensifier during the stance as well as the swing phase, and the force plates were hit correctly. The activity ‘level walking’ required walking straight ahead on the floor; ‘stair descent’ included walking down three 0.18 m steps; ‘sit-to-stand-to-sit’ was performed as a single task without support from the upper extremities.

### 2.3. Motion-capture system and ground reaction forces

A three-dimensional motion analysis system using 12 MX40 motion-capture cameras (Vicon MX system, Oxfords Metrics Group, UK) was employed to capture the movement of a marker attached to the sternum in order to establish the start and end events of the sit-to-stand-to-sit task, with a marker velocity of either greater than or less than 0.02 m s^−1^ used as the threshold criteria.

Ground reaction forces were measured using six force plates mounted in the floor and two mobile force plates mounted in the stair steps (Kistler, Instrumentation, Winterthur, Switzerland) to determine the heel strike and toe-off events for level walking and stair descent, with a threshold of 25 N. All force plates were decoupled from the surrounding floor in order to ensure that the force acquisition was not disturbed by the moving fluoroscope.

### 2.4. Moving fluoroscope

The moving fluoroscope [11,12,22] at the Institute for Biomechanics, ETH Zürich, was used to track the joint motion and image the tibio-femoral implants throughout several consecutive cycles of level walking and stair descent as well as during the sit-to-stand-to-sit task. The image capture was performed using a modified BV Pulsera videofluoroscopy system (Philips Medical Systems, Switzerland) with a field of view of 30.5 cm, pulsed image acquisition rate of 25 Hz, 8 ms radiation pulse-width, 1 ms image shutter time and an image resolution of 1000 × 1000 pixels with a greyscale resolution of 12 bits. The system has previously been used to analyse joint kinematics in patients after total knee and ankle arthroplasty *in vivo* [11,12,38–41].

### 2.5. Data processing

**Figure 1.**
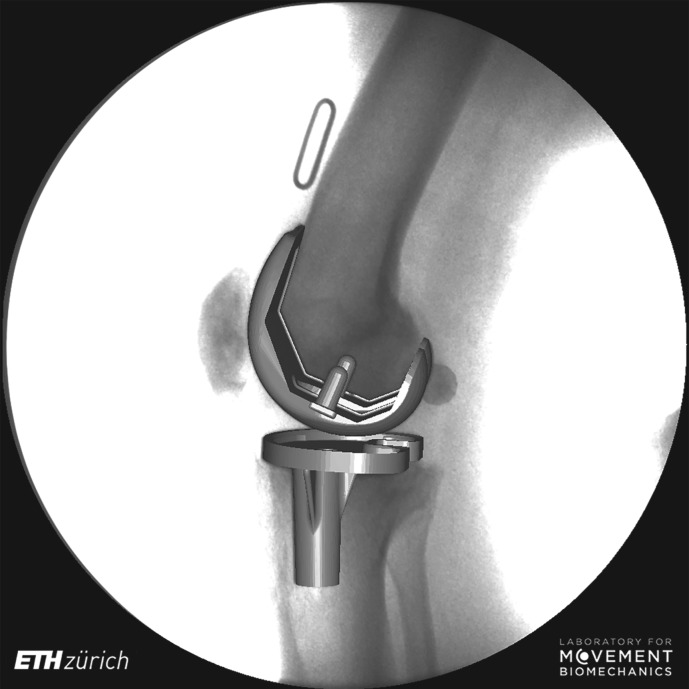
Exemplary instant of three-dimensional tibial and femoral components registered to the two-dimensional fluoroscopic image. The two-dimensional/three-dimensional registration of all images allows the internal joint kinematics to be determined over the complete cycles of functional activities.

Distortion correction of the videofluoroscopic images was performed using an optimization algorithm to correct for local distortions based on images of a reference grid [11,41]. The projection parameters of the videofluoroscopic system (focal distance, and location of the principle point in the image plane) were determined by least-squares optimization using five images of a calibration tube [11,41]. Three-dimensional CAD models of the implant components were then fitted to the two-dimensional fluoroscopic images (figure 1) using a registration algorithm based on the approach presented by Burckhardt and co-workers [42]. Root mean square registration errors using this process have been reported to be less than or equal to 0.25° for all rotations, 0.3 mm for in-plane and 1.0 mm for out-of-plane translations [22].

### 2.6. Tibio-femoral kinematics

**Figure 2.**
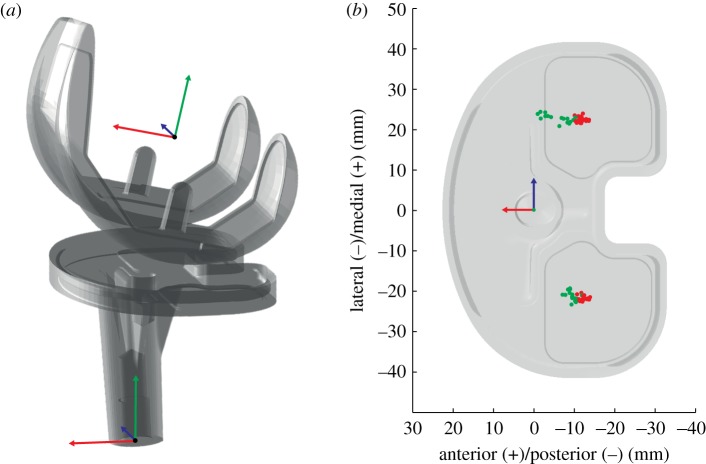
Implant coordinate systems for the femoral and tibial components (*a*) and nearest points for stance (red) and swing (green) phase for an exemplary trial of stair descent presented in the coordinate system of the tibial component (*b*).

Relative tibio-femoral rotations were determined using the joint coordinate system presented by Grood & Suntay [43], based on the local femoral and tibial implant coordinate systems (figure 2, left). Translations of the femoral condyles relative to the tibial component were described using the weighted mean of the 10 nearest points of each femoral condyle to the upper plane of the tibial component. The positions of these nearest points were presented in the coordinate system of the tibial component, thus representing the motion of the femur relative to the tibia (figure 2, right). To interpret joint kinematics, the internal tibial rotation would therefore result in anterior translation of the medial, and/or posterior translation of the lateral, nearest femoral point(s) on the tibia. All kinematic trials were then normalized to one gait cycle.

### 2.7. Statistics

A total of eight linear mixed-model analysis of variances (ANOVAs), with the subject as a random effect, were conducted to comprehensively analyse tibio-femoral kinematics. Specifically, five mixed-model ANOVAs were performed to test the effects of the task on ranges of tibio-femoral rotations and A–P translations of the condyles. Here, task dependency was investigated with rotational (flexion/extension, internal/external, ab/adduction) and translational (medial A–P, lateral A–P) ranges of motion (RoMs) as dependent variables and task as the independent variable with four different levels (level walking, stair stair descent, sit-to-stand, stand-to-sit) for each of those kinematic parameters. Two mixed-model ANOVAs were performed to test the effects of phases of task (e.g. loaded or unloaded phases) on a range of A–P translation of the medial and lateral condyles. In order to analyse phase-dependency, the range of A–P translation of the condyle was the dependent variable, while task with four levels (level walking, stair descent, sit-to-stand, stand-to-sit) and phases of task with two levels (stance and swing phases) were the independent variables. One mixed-model ANOVA was conducted to test the effects of loading sites (e.g. medial or lateral condyles) on a range of A–P translation. The dependency on loading sites was tested with translation range in A–P as the dependent variable and task with four levels (level walking, stair stair descent, sit-to-stand, stand-to-sit) and loading sites with two levels (medial or lateral condyles) as the independent variables. Post hoc comparisons were conducted using the least significant differences (LSD) approach and significance levels were adjusted for multiple comparisons using Bonferroni correction. All ANOVAs were conducted in SPSS (SPSS 23, IBM, USA).

In order to analyse the effects of task dependency on A–P translation at specific flexion angles one-dimensional statistical parametric mapping (SPM) approach was used [44]. One-way ANOVA was performed using the open-source toolbox SPM-1D (Todd Pataky 2017, v. M.0.4.5), with the region of interest defined as the full ranges of flexion angles that are involved in each activity. Here, as loading conditions were totally different between the two phases (stance versus swing) of the gait activities, the two phases were treated as separate tasks. Thus, within the SPM approach, one-way ANOVA was performed with A–P translation as the dependent variable and task with six levels (level walking stance, level walking swing, stair descent stance, stair descent swing, sit-to-stand and stand-to-sit) as independent variables. Post hoc comparisons were conducted using two-sample *t*-tests and significance levels were adjusted for multiple comparisons using Bonferroni correction.

## 3. Results

**Figure 3.**
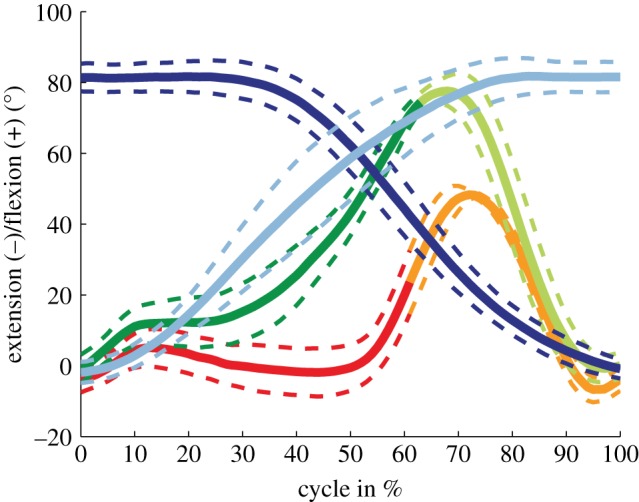
Tibio-femoral flexion is presented for all tasks, including mean and SD over all subjects. Loaded and unloaded activity phases are shown, respectively, for level walking (red/orange), stair descent (green/light green), sit-to-stand (dark blue) and stand-to-sit (light blue).

**Figure 4.**
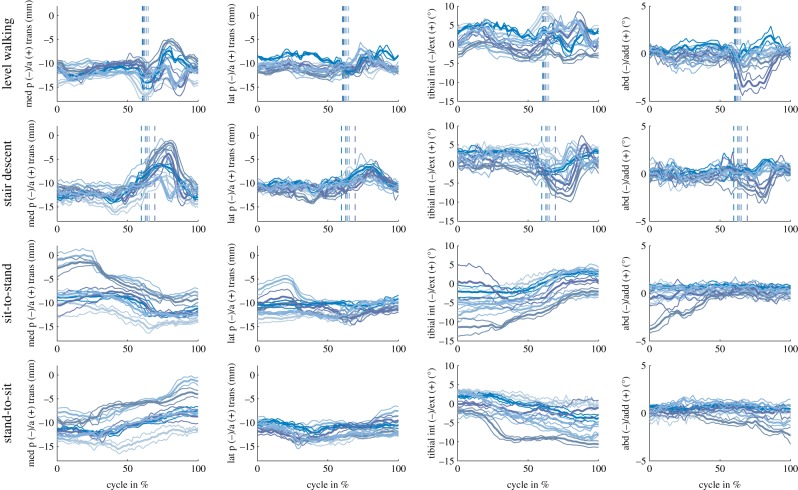
Tibio-femoral kinematics are shown for the activities of level walking, stair descent, sit-to-stand and stand-to-sit for each single subject, represented by the different colours, including the mean and SD over all repetitions. The columns are presented in % cycle and show the A–P translation of the medial and lateral condyles, as well as the tibial internal/external rotation, and joint ab/adduction. For level walking and stair descent, the dashed lines indicate the mean time point of toe-off for each subject.

The mean ranges of joint flexion over all subjects were similar for stair descent (83.9 ± 6.3°), sit-to-stand (83.3 ± 5.6°) and stand-to-sit (84.8 ± 5.0°) (table 1; figure 3), but significantly lower for level walking (58.4 ± 4.1°). No differences between the tasks could be found in the mean range of internal/external rotation or ab/adduction (table 1; figure 4). Mean toe-offs were observed at 62.1 ± 1.7% of the level walking cycle and at 64.1 ± 3.2% of the stair descent cycle.

**Table 1.** Tibio-femoral rotations and A–P translation range for all tasks. For level gait and stair descent, the stance and swing phase were presented separately. Mean and standard deviation over all subjects. Flex/ex: flexion/extension, abd/add: abduction/adduction, int/ext: internal/external tibial rotation, st: stance phase, sw: swing phase, med: medial, lat: lateral, A–P: anterio–posterior.

rotation (°)	flex/ex	abd/add	int/ext	flex/ex (st)	abd/add (st)	int/ext (st)	flex/ex (sw)	abd/add (sw)	int/ext (sw)
level walking^t1^	58.4 ± 4.1^*t2,t3,t4^	3.1 ± 0.9	8.2 ± 1.6	29.5 ± 4.2	2.0 ± 0.5	5.9 ± 1.1	57.6 ± 3.6	2.8 ± 0.9	6.8 ± 1.4
stair descent^t2^	83.9 ± 6.3^*t1^	3.4 ± 0.7	9.2 ± 2.0	75.1 ± 6.4	2.8 ± 0.3	5.9 ± 1.7	82.8 ± 5.9	2.5 ± 0.9	8.4 ± 2.4
sit-to-stand^t3^	83.3 ± 5.6^*t1^	2.3 ± 1.1	8.7 ± 1.7						
stand-to-sit^t4^	84.8 ± 5.0^*t1^	2.2 ± 0.7	7.7 ± 2.0						

translation (mm)	med A–P	lat A–P	med A–P (st)	lat A–P (st)	med A–P (sw)	lat A–P (sw)			
level walking^t1^	6.4 ± 1.2^*t2^	4.9 ± 0.9	3.6 ± 0.9^*p^	3.0 ± 0.6^*p^	6.1 ± 1.3^*p^	4.4 ± 1.0^*p^			
stair descent^t2^	9.4 ± 2.6^*t1,t4,c^	6.5 ± 1.7^*c^	5.0 ± 1.0^*p^	3.7 ± 0.7	8.4 ± 2.3^*p^	4.9 ± 1.2			
sit-to-stand^t3^	7.0 ± 2.0^*c^	4.7 ± 1.7^*c^							
stand-to-sit^t4^	6.5 ± 1.4^*t2^	4.5 ± 0.9							

*t#: significantly different from task ^t#^, based on the adjusted level of significance of *α* = 0.008 (example: *t2 means significantly different to stair descent).

*p: significant difference between stance and swing phase, based on the adjusted level of significance of *α* = 0.025.

*c: significant difference between the medial and lateral condyle, based on the adjusted level of significance of *α* = 0.013.

In general, when tibio-femoral translations and rotations were examined (figure 4), mean and standard deviations of the individual subjects exhibited repeatable individual motion characteristics, indicating small variability between the trials within each single subject, but large inter-subject variations were observed. For example, one subject exhibited a clearly distinct pattern of ab/adduction during the swing phase of level gait compared to the group.

The mean range (difference between minimal and maximal value) of medial condylar A–P translation exhibited a clear task dependency across all subjects (table 1). The mean ranges of A–P translation for the medial condyle were: 6.4 ± 1.2 mm (level walking), 9.4 ± 2.6 mm (stair descent), 7.0 ± 2.0 mm (sit-to-stand) and 6.5 ± 1.4 mm (stand-to-sit); and for the lateral condyle: 4.9 ± 0.9 mm (level walking), 6.5 ± 1.7 mm (stair descent), 4.7 ± 1.7 mm (sit-to-stand) and 4.5 ± 0.9 mm (stand-to-sit). Here, a significantly larger mean range of A–P translation was observed on the medial condyle during stair descent compared to level walking and stand-to-sit. No significant differences between the tasks could be found for the mean range of A–P translation on the lateral condyle. The ranges of A–P translation observed over the full cycles across all subjects were significantly larger for the medial than for the lateral condyle. When activity phases were considered, the mean range of A–P translation for the unloaded swing phases of level walking and stair descent was significantly larger than for the loaded stance phases (table 1). The single greatest posterior and anterior translations of the medial femoral condyle relative to the tibia were −17.9 mm at 60% during a cycle of level walking and 1.6 mm at 18% of a sit-to-stand cycle (in different subjects). The corresponding greatest translations of the lateral femoral condyle were −15.5 mm at 40% during stand-to-sit and −2.6 mm at 73% during stair descent.

**Figure 5.**
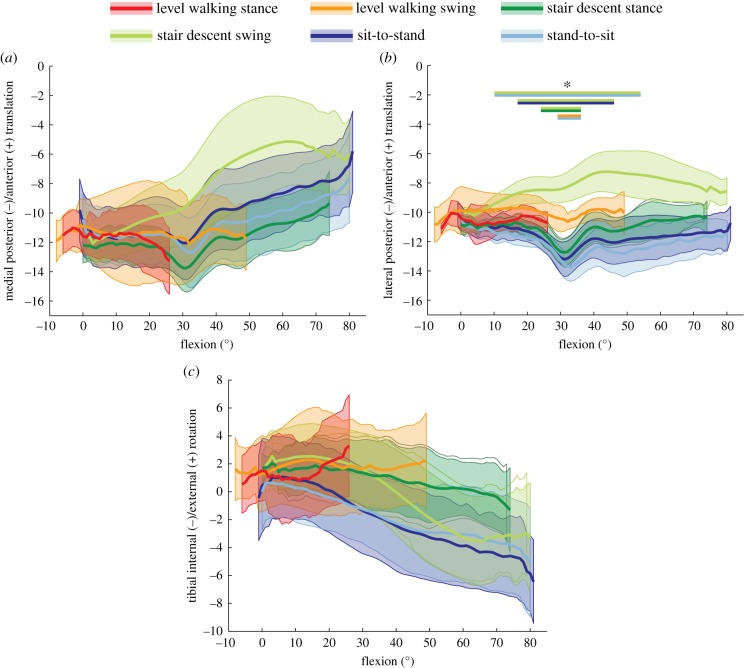
A–P translation for the medial (*a*) and lateral (*b*) condyles, as well as tibial rotation (*c*) are shown as a function of the flexion angle. The tasks level walking, stair descent, sit-to-stand and stand-to-sit are presented as the mean of all six subjects, with the group SD shown as transparent. Note that an anterior translation of the nearest point on the medial condyle and/or posterior translation on the lateral condyle represents an internal rotation of the tibial component. Asterisk (*): significant differences, found between the tasks for certain ranges of flexion, were indicated with bars in the colours of the respective tasks with an adjusted level of significance of *α* = 0.0033.

**Figure 6.**
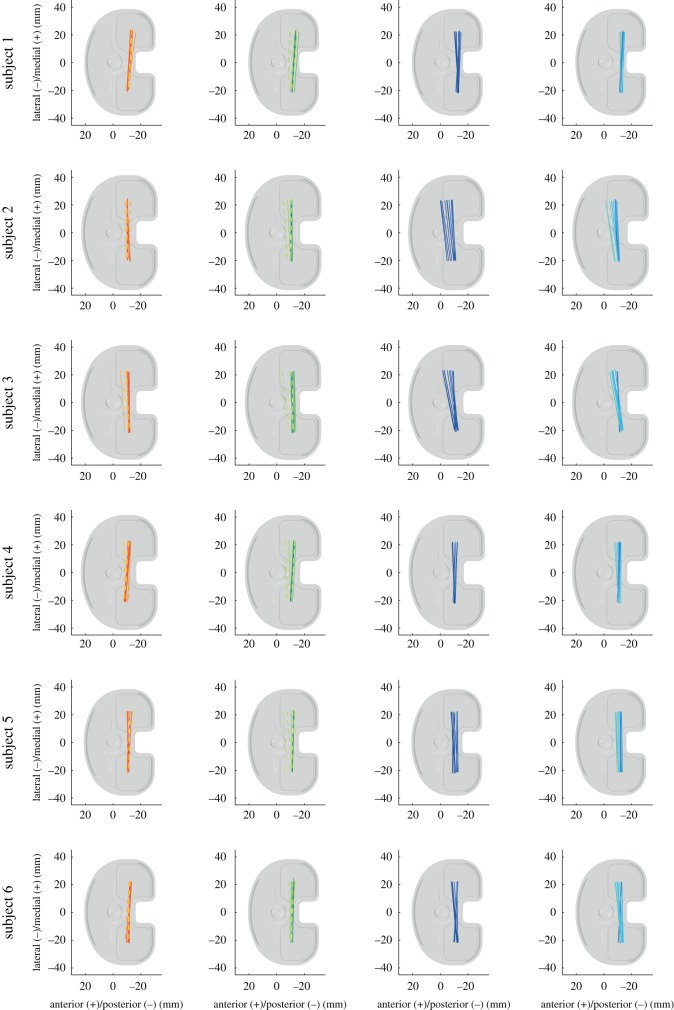
Average positions across all repetitions of the femoral component, represented by lines connecting the nearest points of the medial and lateral condyles relative to the tibial tray for specific time points (dark to bright) during the cycles of level walking (red tones), stair descent (green tones), sit-to-stand (dark blue tones) and stand-to-sit (bright blue tones). Solid lines represent the loaded stance phase and dotted lines the unloaded swing phase.

**Figure 7.**
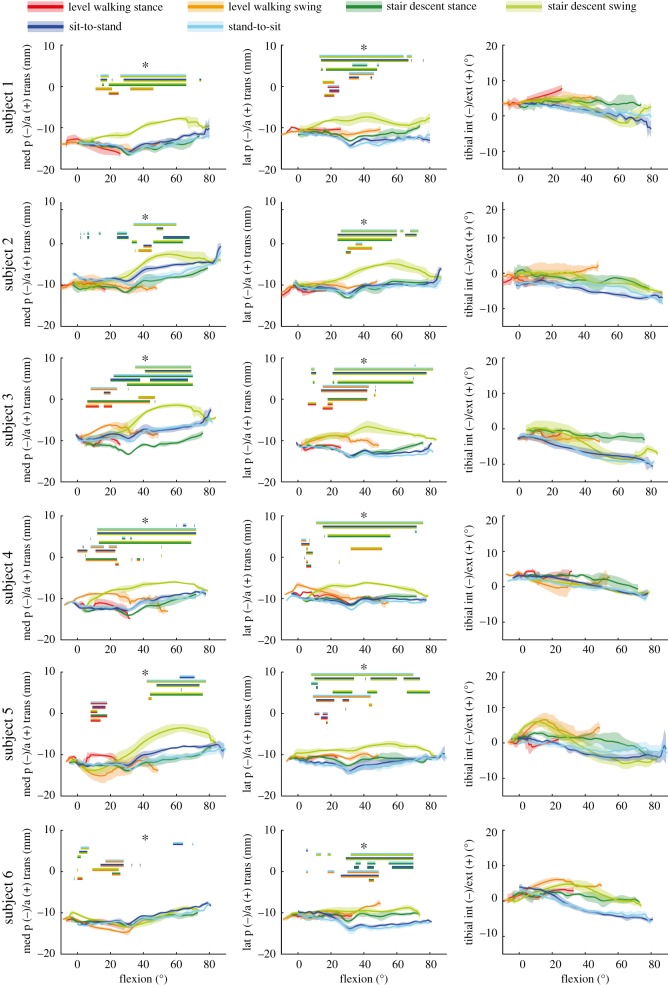
A–P translation for the medial and lateral condyles, as well as tibial rotation are shown as a function of the flexion angle. The tasks level walking, stair descent, sit-to-stand and stand-to-sit are presented for each of the six subjects as the mean of all repetitions, with the subject SD shown as transparent. Asterisk (*): significant differences, found between the tasks for certain ranges of flexion, were indicated with bars in the colours of the respective tasks with an adjusted level of significance of *α* = 0.0033.

In order to establish whether the observed kinematic differences between tasks were simply a function of flexion angle, the flexion dependent A–P translations and internal/external rotations for the different tasks were examined. On average, the lateral A–P translations during the swing phase of stair descent differed significantly from the loaded stance phase (for 24° to 36° flexion), as well as from the sit-to-stand (for 17° to 46° flexion) and stand-to-sit (for 10° to 54° flexion) tasks (figure 5). In addition, the swing phase of level walking showed significant differences to the stand-to-sit task for a certain range of flexion (29° to 36°). Looking at the mean tibial rotation with increasing flexion, a linear increase in the internal tibial rotation was observed for the loaded stance phase of stair descent, as well as during the two sitting tasks. During the unloaded swing phase of stair descent, tibial internal rotation also increased with increasing flexion, but not in a linear manner. However, no increase in the internal rotation was observed with increasing flexion in either phase of level walking. Owing to the large variation between subject kinematics (figures 4 and 6), especially for the medial condyle, task dependency was also investigated on an intra-subject basis over the flexion series (figure 7). Significant task dependency of the loaded phases was found for all subjects between gait activities and the two sitting tasks. When the loaded stance and the unloaded swing phases were compared, all subjects exhibited significant differences when performing stair descent, and two out of six during level gait. Furthermore, four out of six subjects showed a significant difference in A–P translation at the same flexion angles between the movement directions in the two sitting tasks.

Finally, local minima were observed at approximately 30° flexion in most of the A–P translation–flexion curves for different tasks (figure 5), which were more prominent during loaded task phases.

## 4. Discussion

To improve functionality and quality of life, as well as support independent living, joint replacement with a TKA aims to relieve pain and restore the function of the knee joint throughout daily living. While numerous studies have investigated joint movement, a fundamental understanding of tibio-femoral kinematics during dynamic and continuous functional activities of daily living remains lacking, mainly due to the limitations of static imaging modalities [15,45] or the soft tissue artefacts associated with skin-marker based techniques [5]. As a result, the subtle kinematic differences caused by different activities, including different flexion angles, muscular activity, dynamic loading conditions (e.g. impact at heel strike and toe-off), remain generally unknown. The aim of this study was therefore to determine whether knee joint kinematic behaviour after total joint replacement is dependent upon the investigated task and loading conditions. Using dynamic videofluoroscopy [22], we observed that tibio-femoral kinematics were not simply flexion dependent, but rather varied between level walking, stair descent, sit-to-stand and stand-to-sit.

For the first time, the kinematics of the PFC Sigma Curved CR fixed-bearing TKA (DePuy Synthes, Johnson and Johnson) have been evaluated for several consecutive cycles of functional activities, including loaded stance and unloaded swing phases. Here, a comparison of stair descent and the two sitting tasks at the same flexion angles but with different moving directions (figure 5), has clearly demonstrated that an activity-dependent A–P translation–flexion coupling exists. In this respect, it seems that loading and unloading of the implant, together with the movement dynamics and muscular activation patterns, play crucial roles in governing the relative motion within the joint. Additionally, differences in implant movement characteristics, even between the phases of similar gait activities (e.g. level walking and stair descent) at the same joint flexion angles (figure 5), further highlight the importance of analysing whole gait cycles. Here, a complete understanding of the combination of loading/unloading together with the changes in movement directions, might, therefore, be critical for implant design in order to successfully avoid soft tissue overloading [46] but also provide sufficient joint stability for enhanced patient satisfaction [47,48]. For a more detailed analysis of the influence of different loading characteristics on the kinematic parameters, the three components of the ground reaction force could be correlated with the kinematic parameters in a future study. In our study, it was interesting to observe a clear perturbation in the kinematic behaviour during the loaded activity phases, characterized by a local minimum in the A–P translation at 30° flexion (figure 5). This characteristic is in agreement with observations of other studies [49–51] and plausibly corresponds with the change in femoral radius of the PFC Sigma CR implant, and this specific feature could therefore be modulated, controlled or even removed with a different implant design. Interestingly, this feature was much less prominent in the unloaded phases, where the geometry seems to have less impact on kinematic guidance.

The small range of joint flexion involved in level walking has to be considered in the interpretation of the results. The typical local minimum in joint flexion during the stance phase of level walking was not observed during stair descent (figure 3), which is in agreement with the findings of former skin marker studies on healthy knees [52,53]. Moreover, the range of axial rotation occurring during the stance phase of level gait was comparable to the study of Banks & Hodge [18], as well as the study of Schmidt and co-workers [20]. However, the additional access to the motion during the swing phase in our study has resulted in an overall larger range of axial rotation. Banks & Hodge [18] found a significant difference in axial rotation between treadmill walking and a step-up exercise, whereas the results of our study indicate that only flexion/extension ranges of motion exhibited a task dependency, while transverse and frontal plane ranges of motion did not differ. Specifically for the PFC Sigma CR implant, Schmidt *et al*. [20] reported a smaller A–P translation range of the medial condyle (−5.4 mm at heel strike to −6.7 mm at 33% stance phase) but slightly larger translation on the lateral side (−3.8 mm at heel strike to −7.8 mm at toe-off) for discrete time points during the stance phase of walking, compared to our study. For the sitting tasks, larger values especially for the medial and lateral condyle were found compared to deep knee bend up to 90° flexion performed in other studies with a PFC Sigma CR implant [54]. It therefore seems that the additional freedom offered by the moving fluoroscope, which includes not only the loaded stance phase, as in other studies, but also the swing phases of movement and the associated changes in accelerations, movement direction, muscle activity, ground impact, etc., is necessary before an encompassing understanding of the joint motion can be achieved.

In order to compare the results of knee kinematic studies in an objective manner, as well as ensure correct clinical interpretation, the method for kinematic data analysis must be considered with care. Here, the use of a femur fixed geometric axis approach instead of an instantaneous nearest point approach is known to change the interpretation of the A–P characteristics [33,34,55]. Such sensitivities could be especially important when considering the PFC Sigma CR implant investigated in our study, which has two different femoral radii and could therefore lead to crosstalk between flexion and A–P translation. However, the larger A–P translation found for the medial condyle compared to the lateral condyle for all tasks (when using the nearest point technique) indicates that the centre of rotation in the transverse plane in this implant is relatively stable and might be located on the lateral side of the joint, at least for some phases of the activity.

The results of this study revealed a number of interesting aspects relating to the kinematics of this PFC Sigma CR implant. We clearly observed subject-specific movement patterns across the different activities, which were considerably larger than any of the intra-subject differences measured between repetitions. Such differences between subjects indicate that individual anatomical and surgical characteristics, including soft tissue tension [56], component implantation [57] and limb alignment [35] among others, may all play an important role in governing the subject-specific movement patterns. One such characteristic of clinical interest is the possible occurrence of femoral lift-off. Our analyses of ab/adduction suggest that low-level lift-off did indeed occur in 1–2 subjects at specific instances within the functional activities. Whether such kinematic anomalies are indicative of a clinical problem remains to be elucidated, but the ability to detect such small kinematic differences between subjects could suggest that the detailed assessment of internal joint movement (using, e.g. moving fluoroscopic techniques) might be able to support clinical assessments of joint function.

Similar to other studies investigating joint kinematics, the wide-spread extrapolation of our results to, e.g. healthy joints or other implants is restricted by a number of limitations. In particular, the small number of subjects included in our study limits its statistical power for the non-significant differences to represent the more general outcome in a larger population. Furthermore, while the use of a single-plane moving fluoroscope offers considerable advantages in the accurate capture of functional joint kinematics without restrictions due to soft tissue artefacts, the registration of three-dimensional models to two-dimensional images is known to be subject to relatively large out-of-plane errors [11,22]. Such inaccuracies exclude the interpretation of any relative medial–lateral movement of the components. In addition, the extreme accelerations that occur in the human knee joint limit the ability of the moving fluoroscope to track walking activities at speeds beyond that of slow gait [58]. Finally, this study included only TKA subjects with a good clinical outcome. It remains to be investigated whether these results can be extrapolated for understanding joint kinematics in other implants, including TKAs with a bad outcome, or especially whether these results are comparable to the kinematics of healthy knees.

In summary, comparisons between the different tasks and phases within the six subjects investigated in our study showed a clear task dependency but the impact of the task on the underlying kinematics seems to be subject-specific. Differences in dynamics, limb alignment, range of motion, muscle activation or balancing of the ligaments, might well be able to explain these subject-specific characteristics. However, from the data presented within this study, it is clear that the flexion angle alone cannot fully explain tibio-femoral implant kinematics. As a result, it seems that the assessment of complete cycles of the most frequent functional activities of daily living seems to be imperative when evaluating the behaviour of a TKA design *in vivo*.

## Data accessibility

Additional data files of the individual subjects can be publicly accessed here: http://www.movement.ethz.ch/data-repository.
